# Pharmacological Activation of Autophagy Ameliorates Neuromuscular-Associated Defects and Extends Lifespan in a *Caenorhabditis elegans* Model of Spinal Muscular Atrophy

**DOI:** 10.1021/acschemneuro.6c00569

**Published:** 2026-09-02

**Authors:** Saman Rashid, Coral Crespo Gómez, Maria Dimitriadi

**Affiliations:** 3769 University of Hertfordshire, School of Health, Medicine and Life Sciences, Hatfield, England AL10 9AB, U.K.

**Keywords:** Spinal muscular atrophy, autophagy, *C. elegans*, drug screen, neuromuscular function, neurodegeneration

## Abstract

Spinal muscular atrophy (SMA) is a severe neuromuscular disorder caused by homozygous deletions or mutations in the *SMN1* gene, resulting in reduced levels of the ubiquitously expressed survival motor neuron protein and progressive degeneration of lower motor neurons. Although autophagy dysregulation has been implicated in SMA pathology, the therapeutic benefit of modulating this pathway remains controversial. Here, we screened autophagy modulators in a previously characterized *Caenorhabditis elegans* SMA model. We identified that pharmacological activation of autophagy, but not inhibition, significantly improved neuromuscular function, as indicated by two independent functional readouts: pharyngeal pumping and locomotor activity assays. These beneficial effects were validated in an independent severe *C. elegans* SMA mutant allele and were observed in metabolically active and inactive food conditions. Furthermore, autophagy activators significantly extended lifespan of SMA nematodes without affecting controls. Notably, rapamycin and resveratrol increased endogenous SMN-1 protein levels, and improved neuromuscular function was dependent on an intact autophagy pathway. Collectively, our findings support enhancing autophagic activity as a combinatorial treatment strategy for SMA patients.

## Graphical abstract

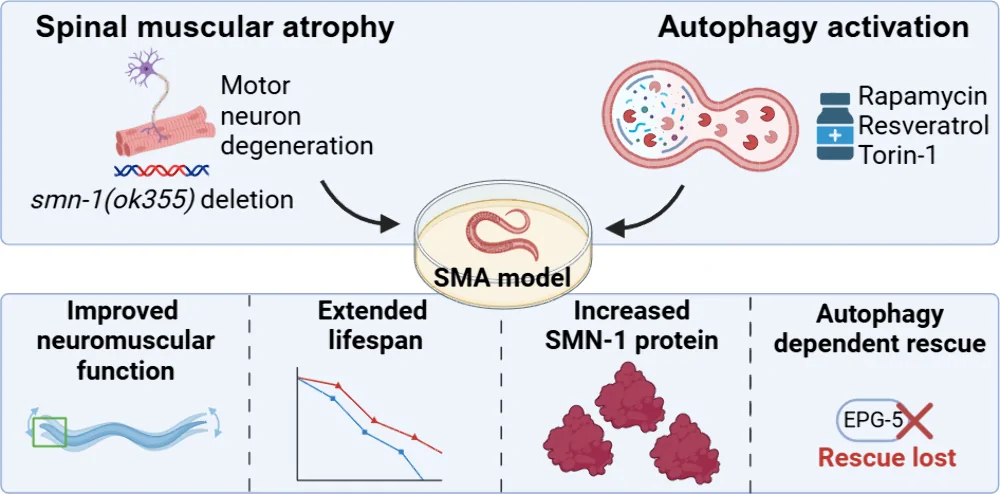

Spinal muscular atrophy (SMA) is a severe autosomal recessive neuromuscular disorder and the leading genetic cause of infant mortality.^1^ SMA is caused by loss of the survival motor neuron 1 (*SMN1*) gene, resulting in reduced levels of the ubiquitously expressed survival motor neuron (SMN) protein.^2^ A nearly identical paralog, *SMN2*, differs from *SMN1* by a few nucleotides, including a critical substitution in exon 7 that disrupts splicing and promotes exon skipping.^3^ Consequently, the majority of *SMN2* transcripts encode a truncated and unstable SMNΔ7 protein, while only approximately 10% produce full-length, functional SMN.^3^ Although *SMN2* copy number modulates disease severity, its expression is insufficient to fully compensate for the loss of *SMN1* function and prevent SMA pathology.^4^

Despite the ubiquitous expression of the SMN protein, motor neurons display a pronounced selective vulnerability to its depletion; however, the molecular and cellular mechanisms underlying this susceptibility are the subject of ongoing investigation. While SMN’s canonical role is in the assembly of small nuclear ribonucleoprotein (snRNP) particles required for pre-mRNA splicing,^5–7^ no single splicing defect has been identified that fully accounts for SMA pathology and progression. Consequently, attention has increasingly focused on the alternative function(s) of the SMN protein, including axonal transport,^8^ neuromuscular junction maturation and function,^9,10^ mitochondrial biogenesis and maintenance,^11,12^ cytoskeletal dynamics,^13,14^ metabolism,^15^ endocytosis^16–19^ and autophagy.^12,20–26^

Autophagy, a highly conserved lysosomal degradation pathway, is dysregulated in SMA and other neurodegenerative diseases.^21,27–29^ The pathway comprises five main steps: (i) induction, (ii) vesicle nucleation, (iii) vesicle elongation, (iv) docking/fusion with the lysosome, and (v) degradation. Progression through these steps is referred to as autophagic flux, which can be disrupted at any stage, compromising turnover of cellular cargo.^30^

Although accumulation of autophagosomes (i.e., double-membraned vesicles that sequester cytoplasmic cargo) and impaired autophagic flux have been observed in SMA, it remains controversial whether autophagy activation or inhibition is ultimately beneficial.^22,24,25^ These apparently divergent findings may, in part, reflect tissue-specific differences in autophagic activity.^26^ For example, the autophagy inhibitor 3-methyladenine was reported to rescue motor neurons and extend lifespan in an intermediate SMA mouse model.^24^ Similarly, inhibition of autophagy via Bcl-XL overexpression or p62 knockdown was observed to rescue SMA-related phenotypes in cultured motor neurons and in *Drosophila* and mouse models of SMA, respectively.^20,25^ Conversely, activators such as urolithin A and rapamycin have been shown to improve mitochondrial health and increase SMN levels in muscles and cultured spinal cord motor neurons.^23,31^ Ultimately, autophagy is a double-edged sword in which both excessive activation and impairment can be detrimental.^32,33^ Accordingly, the therapeutic value of modulating autophagy in SMA remains to be established.

*Caenorhabditis elegans* is a versatile model organism that is used extensively to study a broad range of neurodegenerative diseases, including SMA. *C. elegans* have a single *smn-1* gene orthologous to the human *SMN1*, deletion of which recapitulates key neurodegenerative phenotypes in SMA, including impaired neuromuscular function and reduced lifespan (Figure S1).^34–36^ Here, we used a well-characterized *C. elegans* SMA severe model to evaluate the effect of autophagy modulation on neuromuscular function and lifespan, providing critical insights into combinatorial therapeutic strategies for motor neuron diseases.

To address this, we first investigated the effect of known autophagy modulators on *smn-1­(ok355)* animals, the severe *C. elegans* model of SMA.^34^ We selected four autophagy inhibitors based on their established mechanisms of action and prior relevance to SMA research: (i) 3-methyladenine, an early stage autophagy inhibitor previously evaluated in SMA studies,^24^ (ii) chloroquine and (iii) hydroxychloroquine both of which inhibit late-stage autophagy^37,38^ and (iv) wortmannin, a phosphoinositide 3-kinase inhibitor that suppresses autophagy initiation.^39^ To assess the effects of these autophagy inhibitors on neuromuscular function, we measured the pharyngeal pumping activity as a functional readout. The *C. elegans* pharynx is an organ controlled by its own neuromuscular system used for feeding. Control animals exhibit rhythmic pharyngeal contractions at 200–250 pumps per minute, whereas SMA animals show a marked reduction of 30–50 pumps per minute.^34,36,40^ Screening at the last larval stage — where *smn-1­(ok355)* animals arrest^34^ — revealed that autophagy inhibitors 3-methyladenine, chloroquine, hydroxychloroquine and wortmannin did not improve pharyngeal pumping rates in *smn-1­(ok355)* animals (Figure 1A-D). In fact, hydroxychloroquine reduced pharyngeal pumping in *smn-1­(ok355)* animals at 100 μM (*p = 0.0444*), while pharyngeal pumping in controls was increased at all doses (*p = 0.0097, p = 0.0121 and p = 0.0225*, respectively) (Figure 1C). These findings suggest that autophagy inhibition does not improve pharyngeal pumping defects in *smn-1­(ok355)* animals.

**Figure 1:**
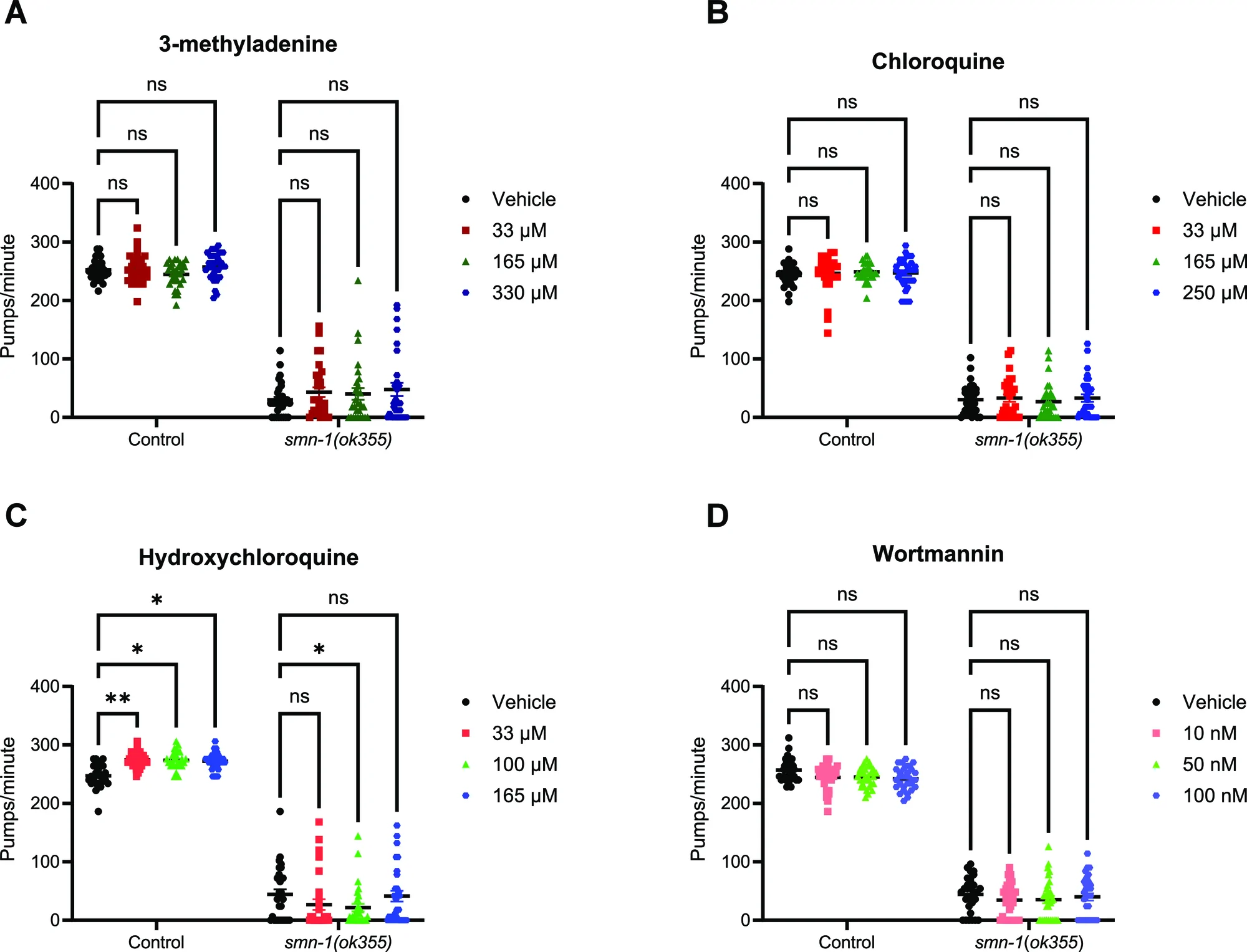
**Autophagy inhibitors do not improve neuromuscular function as assessed by pharyngeal pumping in** ***smn-1­(ok355)*** **animals.** *smn-1­(ok355)* and control animals were reared on NGM plates containing either vehicle or varying concentrations of autophagy inhibitors. Animals were assessed at Day 3 post hatching. Pharyngeal pumping was defined as a complete contraction and relaxation of the grinder. (A) 3-methyladenine had no significant effect at any concentration assessed. (B) Chloroquine had no significant effect at any concentration assessed. (C) Hydroxychloroquine increased pharyngeal pumping in control animals at all concentrations and reduced *smn-1­(ok355)* pharyngeal pumping at 100 μM. (D) Wortmannin had no significant effect at any concentration assessed. Data shown as a scatter plot; error bars represent ± standard error of the mean (SEM); *n* > 25 animals per genotype per concentration across ≥ 3 independent trials; Two-Way ANOVA with Sidak’s multiple comparison test; ns = not significant, *p < 0.05, **p < 0.01.

Next, we selected four autophagy activators based on their established mechanisms and prior relevance to neurodegenerative models: (i) rapamycin and (ii) resveratrol, both previously reported to induce autophagy and evaluated in SMA studies;^25,41–44^ (iii) torin-1, a potent mTOR inhibitor that robustly stimulates autophagy^45^ and (iv) spermidine, a polyamine known to enhance autophagy and clear protein aggregates in *C. elegans*.^46,47^ In contrast to our inhibitory studies, rapamycin, resveratrol, torin-1 and spermidine significantly increased pumping rates in *smn-1­(ok355)* animals (Figure 2A-D). Specifically, rapamycin partially ameliorated pharyngeal pumping defects in *smn-1­(ok355)* animals at all doses (*1 μM, p = 0.0003; 50 μM, p < 0.0001; 100 μM, p < 0.0001*), while reducing control pharyngeal pumps at 50 μM (*p = 0.0140*) and 100 μM (*p < 0.0001*) (Figure 2A). Resveratrol increased pharyngeal pumping in *smn-1­(ok355)* animals at 100 μM and 200 μM (*p < 0.0001*) (Figure 2B) but had no effect on control animals. Torin-1 was capable of partially rescuing *smn-1­(ok355)* pharyngeal pumping at 500 nM (*p = 0.0002*) and reduced control pumping at 1000 nM (*p < 0.0001*) (Figure 2C). Spermidine displayed an ability to increase pharyngeal pumping in *smn-1­(ok355)* animals at all doses (33 μM, *p = 0.0378*; 165 μM, *p = 0.0376*; 330 μM, *p < 0.0001*) and controls at 165 μM and 330 μM (*p < 0.0001*) (Figure 2D). These findings demonstrate that autophagy activators rapamycin, resveratrol and torin-1 increased pharyngeal pumping rates in *smn-1*-deficient animals, while exerting no beneficial effect in controls, indicating specificity to the SMA phenotype (Figure 2A-C). The improvement in neuromuscular function observed following spermidine treatment, previously documented to display neuroprotective effects across multiple organisms including *C. elegans*,^48–50^ was not specific to SMN depletion as comparable effects were detected in control animals (Figure 2D). Consequently, spermidine was not pursued in subsequent analyses. Moreover, the beneficial effects of autophagy activation were recapitulated in an independent severe *C. elegans* SMA allele, *smn-1­(rt248)*. Notably, these effects were observed in the absence of metabolically active food, suggesting that the partial restoration in neuromuscular function was robust across distinct genetic backgrounds and dietary conditions (Figures S2 and S3).

**Figure 2:**
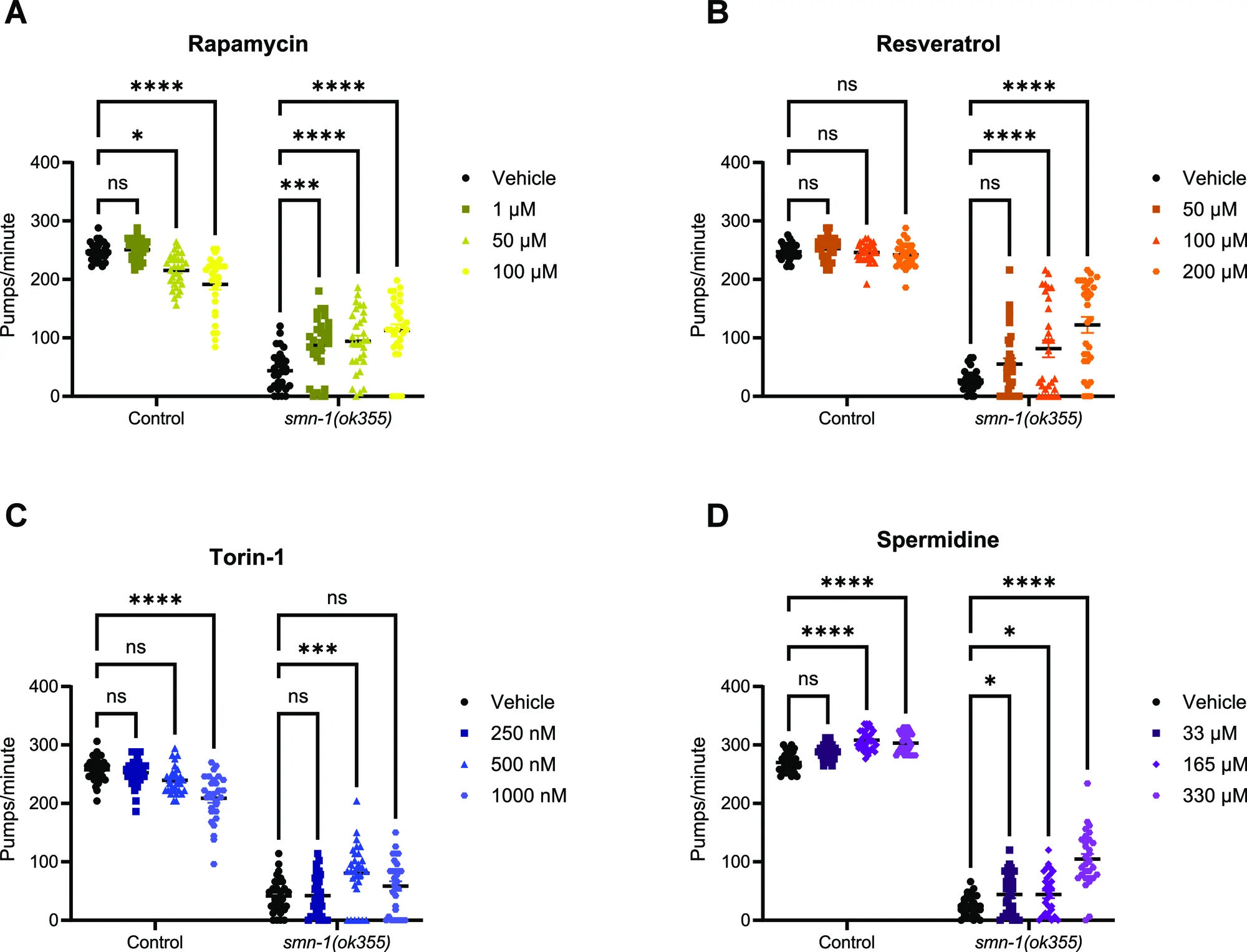
**Autophagy activators improve neuromuscular function by pharyngeal pumping in** ***smn-1­(ok355)*** **animals.** *smn-1­(ok355)* and control animals were reared on NGM plates containing either vehicle or varying concentrations of autophagy activators. Animals were assessed at Day 3 post hatching. Pharyngeal pumping was defined as a complete contraction and relaxation of the grinder. (A) Rapamycin reduced pharyngeal pumping in control animals at 50 μM and 100 μM and increased pharyngeal pumping in *smn-1­(ok355)* animals at all concentrations tested. (B) Resveratrol increased pharyngeal pumping in *smn-1­(ok355)* animals at 100 μM and 200 μM. (C) Torin-1 reduced pharyngeal pumping at 500 nM and 1000 nM in control animals but increased in *smn-1­(ok355)* animals at 500 nM. (D) Spermidine increased pharyngeal pumping in both control and *smn-1­(ok355)* animals. Data shown as a scatter plot; error bars represent ± SEM; *n* > 25 animals per genotype per concentration across ≥ 3 independent trials; Two-Way ANOVA with Sidak’s multiple comparison test; ns = not significant, *p < 0.05, **p < 0.01, ***p < 0.001, and ****p < 0.0001.

To further assess neuromuscular function, we next examined locomotion, an additional neuromuscular phenotype impaired in *smn-1­(ok355)* animals.^34^ Treatment with autophagy activators led to significant improvements in overall animal movement and a reduction in mobility idle time (Figure 3A-E). Rapamycin treatment partially improved locomotion in *smn-1­(ok355)* animals. For example, total distance traveled was significantly increased at 50 μM (*p = 0.0005*) and 100 μM (*p < 0.0001*) (Figure 3A), while mobility idle time was significantly reduced at both these concentrations (*p < 0.0001*) (Figure 3B). In contrast, control animals displayed a significant reduction in distance traveled at both concentrations (50 μM, *p* = 0.0080; 100 μM, *p* < 0.0001), accompanied by a corresponding increase in mobility idle time (*p = 0.0358*). Resveratrol demonstrated consistent improvements in locomotory performance. Specifically, in *smn-1­(ok355)* mutants, total distance traveled was significantly increased at both 100 μM (*p < 0.0001*) and 200 μM (*p < 0.0001*) (Figure 3C), accompanied by a significant reduction in mobility idle time (*p < 0.0001*) (Figure 3D). Torin-1 treatment also produced improvements in *smn-1­(ok355)* locomotory function. Total distance traveled significantly increased at 500 nM (*p = 0.0011*) and 1000 nM (*p = 0.0445*) (Figure 3E), while mobility idle time was significantly reduced at both higher concentrations (500 nM, *p < 0.0001*; 1000 nM, *p = 0.0005*) (Figure 3F). In control animals, total distance traveled was reduced at 1000 nM (*p = 0*.0023) with no changes observed in mobility idle time. Together, these results demonstrate that autophagy activation specifically improves neuromuscular function in *C. elegans* SMA models.

**Figure 3:**
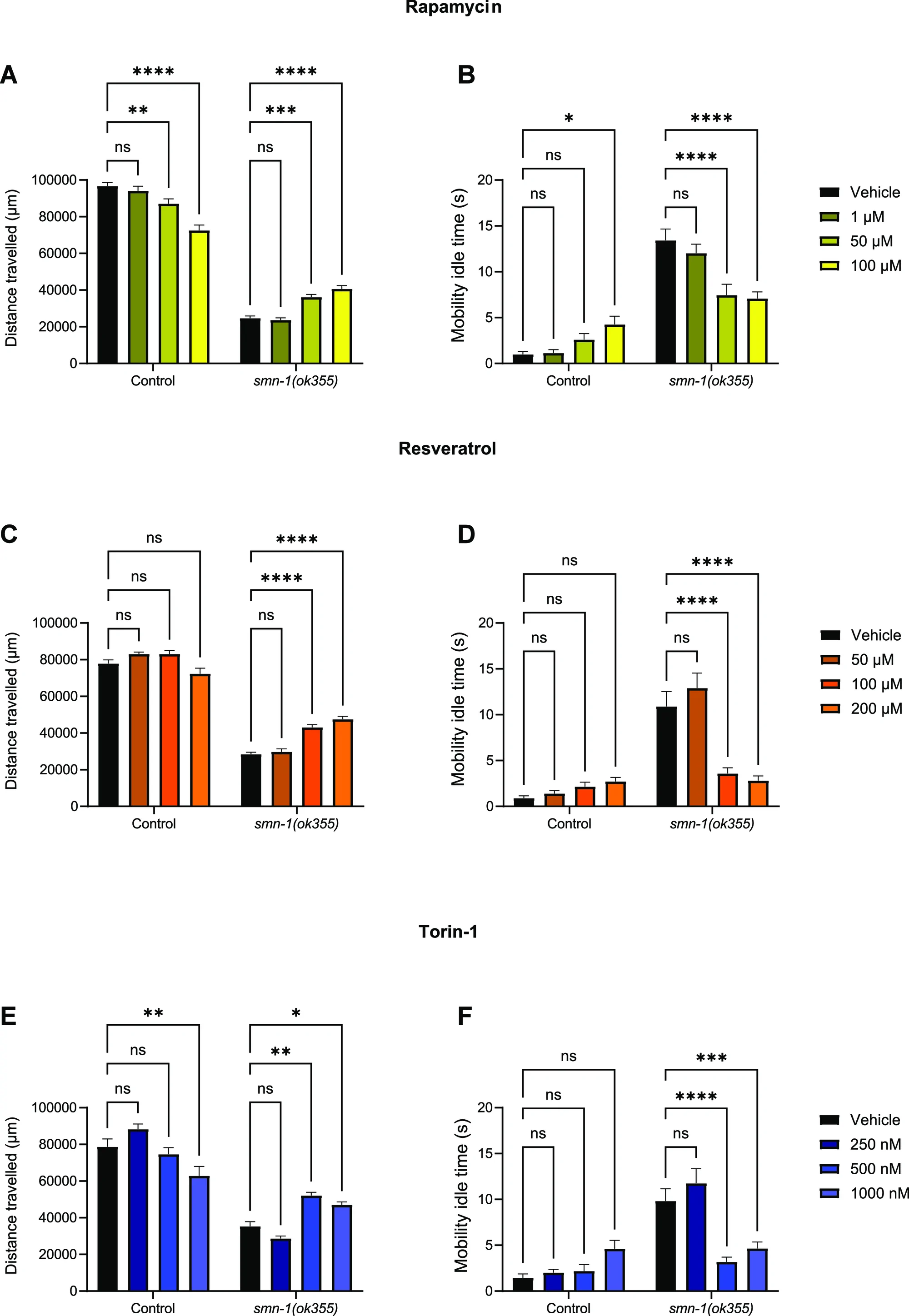
**Autophagy activators alleviate locomotion defects in** ***smn-1­(ok355)*** **animals.** *smn-1­(ok355)* and control animals were reared on NGM plates containing either vehicle or varying concentrations of autophagy activators. Animals were assessed at Day 3 post hatching. (A-B) Rapamycin increased the overall distance traveled and reduced mobility idle time in *smn-1­(ok355)* animals, whereas it elicited the opposite effect in control animals. (C-D) Resveratrol increased the overall distance traveled and reduced mobility idle time in *smn-1­(ok355)* animals. (E-F) Torin-1 increased the overall distance traveled and reduced mobility idle time in *smn-1­(ok355)* animals, whereas it produced the opposite effect observed in controls. Data shown as bar graphs; error bars represent ± SEM; *n* > 25 animals per genotype per concentration across ≥ 3 independent trials; Two-Way ANOVA with Sidak’s multiple comparison test; ns = not significant, *p < 0.05, **p < 0.01, ***p < 0.001, and ****p < 0.0001.

We next investigated whether autophagy activators could affect *smn-1­(ok355)* survival rates. *C. elegans* typically live approximately 21 days under nutrient-rich conditions, whereas *smn-1­(ok355)* animals have a reduced lifespan of approximately 7 days.^34^ Treatment with rapamycin (50 μM, *p = 0.0001;* 100 μM, *p = 0.0486*), resveratrol (*p < 0.0001*) and torin-1 (*p < 0.0001* and *p = 0.0036*) significantly extended the lifespan of SMA nematodes (Figure 4A-C). Notably, treatment with rapamycin did not alter the median lifespan of *smn-1­(ok355)* animals, which remained at 6 days (Figure 4A). However, survival curve analysis revealed a consistent increase in the proportion of *smn-1­(ok355)* surviving animals at successive daily time points following rapamycin treatment compared with vehicle-treated controls. For instance, on day 5, where approximately 50% of vehicle-treated *smn-1­(ok355)* animals remained alive, 73% of nematodes exposed to 50 μM rapamycin remained viable (Figure 4A, Table S1). Resveratrol treatment resulted in a modest increase in median lifespan, from 5 days in vehicle-treated animals to 6 days at both 100 μM and 200 μM concentrations (Figure 4B). Additionally, resveratrol-treated animals maintained a higher survival fraction throughout the assay, with 73% of animals in the 200 μM group surviving to day 5 (Figure 4B, Table S1). Interestingly, torin-1 treatment was associated with an initial reduction in survival, followed by a relative increase in lifespan after day 4. By day 5, 69% of *smn-1­(ok355)* animals treated with 500 nM torin-1 remained viable (Figure 4C, Table S1). Torin-1, like rapamycin, also conferred no change in median lifespan (Figure 4C). Importantly, no lifespan extension was observed in control animals (Figure S4), supporting the specificity of the pro-survival effects in *smn-1­(ok355)* mutants.

**Figure 4:**
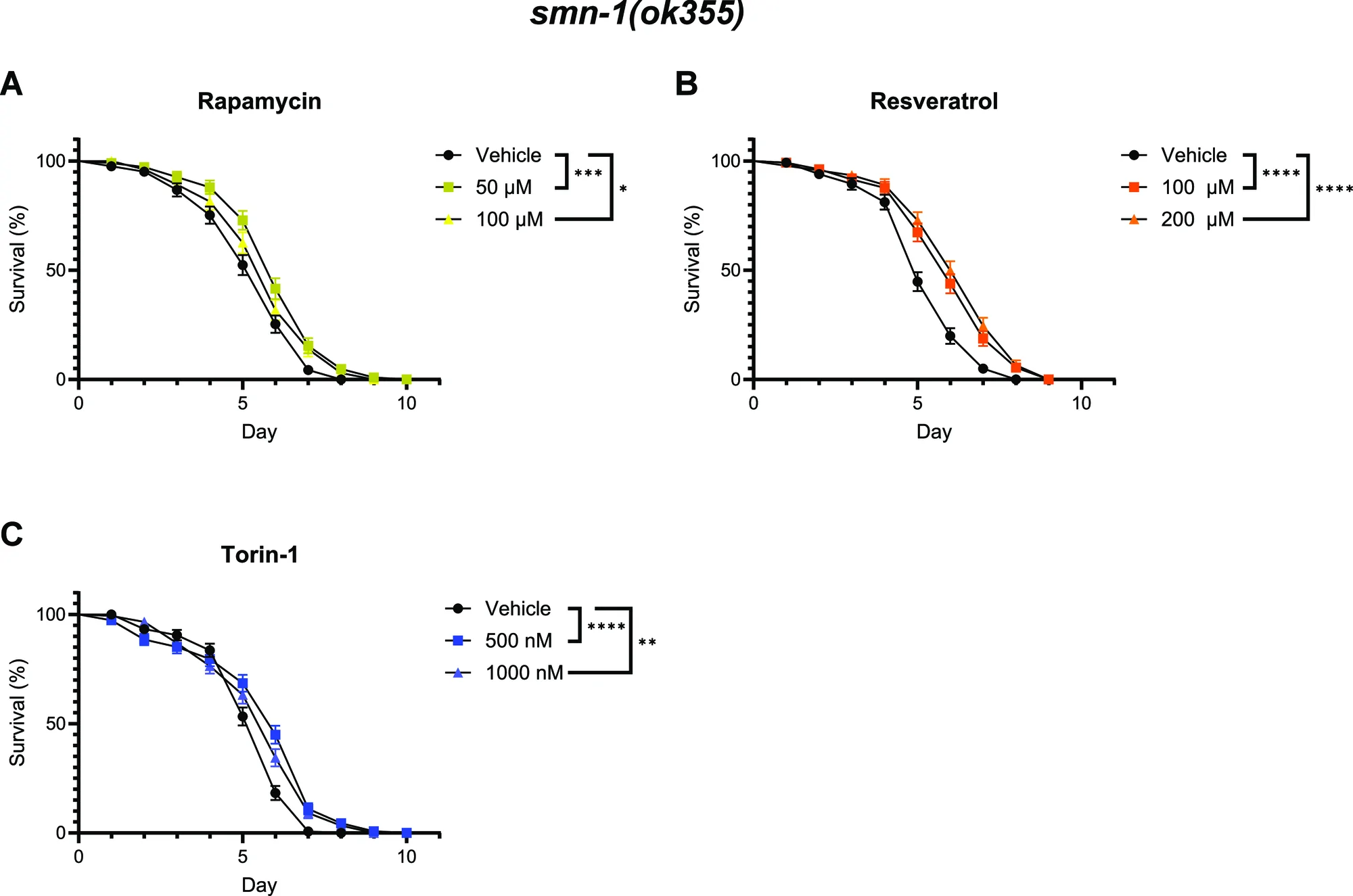
**Autophagy activators increase** ***smn-1­(ok355)*** **lifespan.** *smn-1­(ok355)* were reared on NGM plates containing either vehicle or varying concentrations of autophagy activators. Day 3 animals were transferred to regular NGM plates. Animals were then scored for survival daily and censored for loss, intestinal rupture, internal hatching or crawling off the plate. (A) Rapamycin increased *smn-1­(ok355)* lifespan. (B) Resveratrol increased *smn-1­(ok355)* lifespan. (C) Torin-1 increased *smn-1­(ok355)* lifespan. Data shown as a Kaplan–Meier survival curves; error bars represent the standard error of the survival estimate; ≥90 per concentration across ≥3 independent trials; log-rank test; ns = not significant, *p < 0.05, **p < 0.01, ***p < 0.001, and ****p < 0.0001.

To investigate whether the phenotypic effects of autophagy modulation were associated with changes in SMN-1 protein abundance, we quantified SMN-1 fluorescence intensity in control and *smn-1­(ok355)* animals following treatment with two autophagy inhibitors, 3-methyladenine and chloroquine, and two autophagy activators, rapamycin and resveratrol. Treatment with 3-methyladenine did not significantly alter SMN-1 fluorescence intensity in either control or *smn-1­(ok355)* animals at any concentration examined (Figure 5A). Similarly, chloroquine had no significant effect in control animals (Figure 5B). However, chloroquine significantly reduced SMN-1 fluorescence intensity in *smn-1­(ok355)* animals at both 165 μM and 250 μM (*p < 0.001*; Figure 5B). In contrast, treatment with the autophagy activators increased SMN-1 fluorescence intensity in *smn-1­(ok355)* animals. More specifically, rapamycin significantly increased SMN-1 fluorescence at both 50 μM and 100 μM in *smn-1­(ok355)* animals (*p < 0.0001*; Figure 5C). Interestingly, the same concentrations significantly reduced SMN-1 fluorescence in control animals (*p < 0.0001*), indicating a genotype-dependent effect. Resveratrol also significantly increased SMN-1 fluorescence in *smn-1­(ok355)* animals at 100 μM (*p < 0.01*) and 200 μM (*p < 0.0001*; Figure 5D). Unlike rapamycin, resveratrol increased SMN-1 fluorescence in control animals at 100 μM (*p < 0.0001*) and 200 μM (*p < 0.001*). Collectively, these results demonstrate that autophagy activators rapamycin and resveratrol increased SMN-1 protein abundance in *smn-1­(ok355)* animals, whereas the autophagy inhibitors either had no effect or, in the case of chloroquine, further reduced SMN-1 abundance. The association between increased SMN-1 fluorescence and improved *smn-1* neuromuscular phenotypes suggests that the beneficial effects of autophagy activation may be mediated, at least in part, through increased SMN-1 protein abundance. However, the compound- and genotype-dependent responses observed in control animals indicate that the effects of autophagy modulation are unlikely to result from a uniform increase in SMN-1 levels alone.

**Figure 5:**
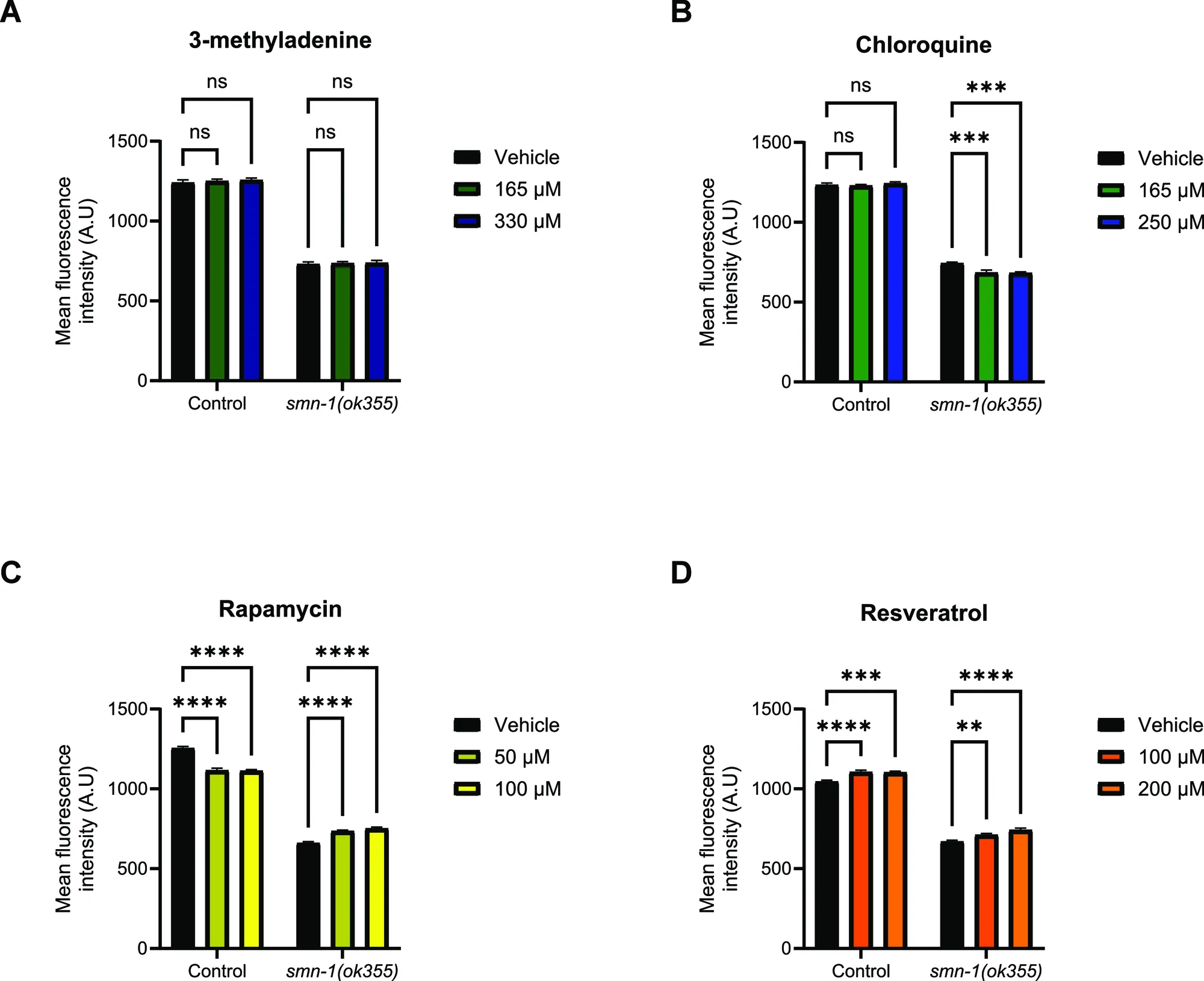
**Autophagy modulators differentially alter SMN-1 protein abundance in control and** ***smn-1­(ok355)*** **animals.** Control and *smn-1­(ok355)* animals were reared on NGM plates containing vehicle or the indicated concentrations of selected autophagy modulators. SMN-1 protein abundance was assessed by quantification of mean fluorescence intensity in L4 stage animals. (A) 3-methyladenine did not significantly alter SMN-1 fluorescence intensity in either control or *smn-1­(ok355)* animals. (B) Chloroquine did not alter SMN-1 fluorescence in control animals but significantly reduced fluorescence intensity in *smn-1­(ok355)* animals at 165 μM and 250 μM. (C) Rapamycin reduced SMN-1 fluorescence intensity in control animals but increased fluorescence in *smn-1­(ok355)* animals at 50 μM and 100 μM. (D) Resveratrol increased SMN-1 fluorescence intensity in both control and *smn-1­(ok355)* animals. Data are presented as mean ± SEM; *n* > 25 animals per genotype per concentration across ≥ 3 independent trials; Two-Way ANOVA with Sidak’s multiple comparison test; ns = not significant, *p < 0.05, **p < 0.01, ***p < 0.001, and ****p < 0.0001.

To determine whether the beneficial effects of autophagy activators were functionally dependent on an intact autophagy pathway, we assessed the effects of rapamycin and resveratrol in *epg-5­(tm3425)* mutants and created *smn-1­(ok355);epg-5­(tm3425)* double mutant animals. *epg-5* encodes a core autophagy regulator required for proper autophagic flux,^51,52^ and its loss provides a genetic approach to test whether the *smn-1* phenotypic improvements conferred by these compounds depend on autophagy. Consistent with our earlier findings, rapamycin treatment improved pharyngeal pumping, increased total distance traveled, and reduced mobility idle time in *smn-1­(ok355)* animals (Figure 6A-C). However, these beneficial effects were abolished in *smn-1­(ok355);epg-5­(tm3425)* double mutants, in which rapamycin failed to improve any of the neuromuscular readouts assessed. Similarly, resveratrol significantly ameliorated pharyngeal pumping and locomotory defects in *smn-1­(ok355)* animals, but these improvements were not observed in the *smn-1­(ok355);epg-5­(tm3425)* genetic background (Figure 6D-F). Notably, neither rapamycin nor resveratrol produced a comparable rescue in *epg-5­(tm3425)* single mutants or in the *smn-1­(ok355);epg-5­(tm3425)* double mutants, indicating that the beneficial effects observed in *smn-1­(ok355)* animals require intact autophagy. Together, these findings provide genetic evidence that the functional rescue conferred by rapamycin and resveratrol is autophagy-dependent, supporting the conclusion that pharmacological activation of autophagy underlies the improvement in SMA-associated phenotypes.

**Figure 6:**
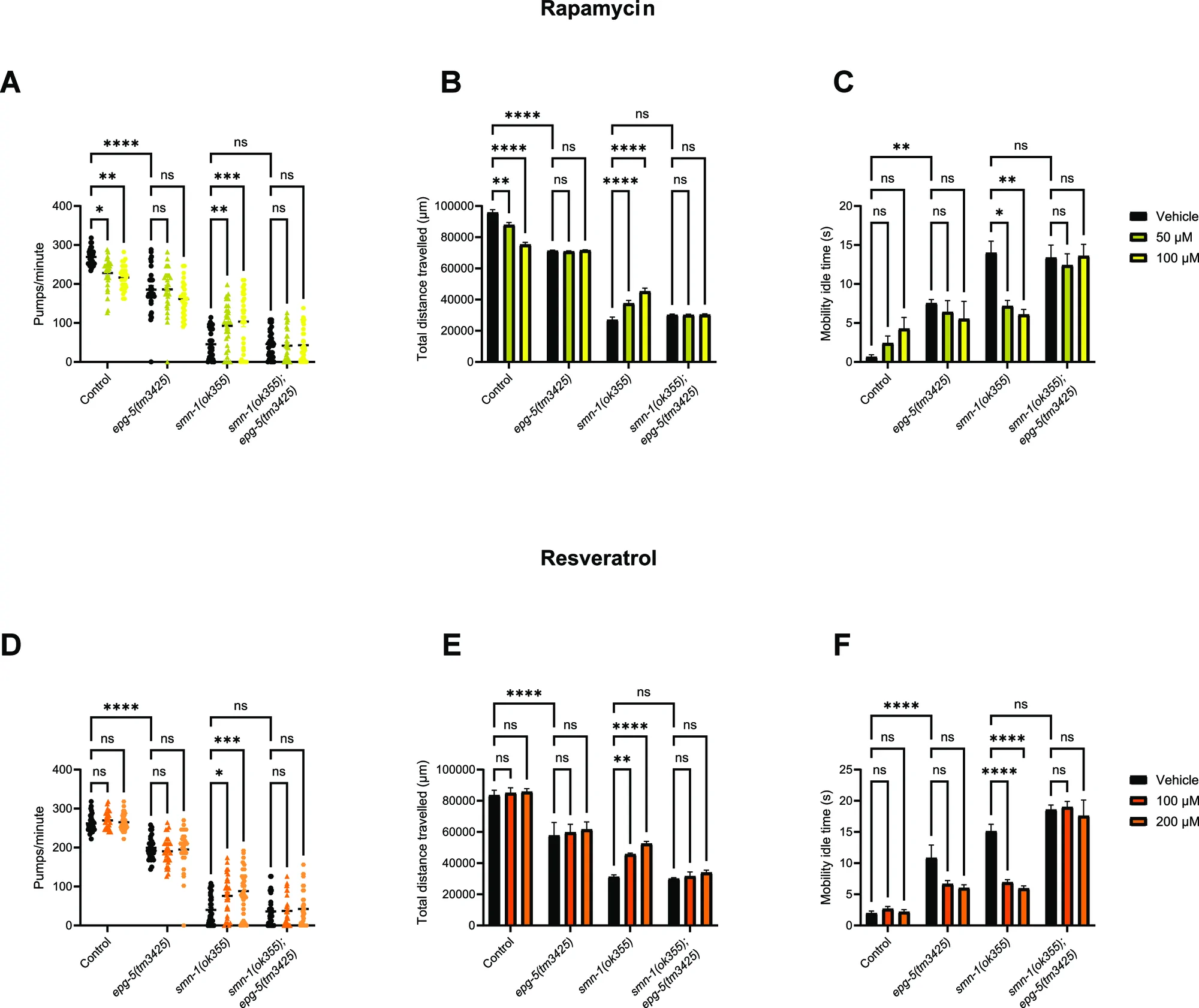
**Functional rescue by autophagy activators requires intact autophagy.** Control, *epg-5­(tm3425), smn-1­(ok355)* and *smn-1­(ok355);epg-5­(tm3425)* animals were reared on NGM plates containing vehicle or the indicated concentrations of rapamycin or resveratrol. Animals were assessed at day 3 posthatching. (A-C) Rapamycin improved pharyngeal pumping, increased total distance traveled and reduced mobility idle time in *smn-1­(ok355)* animals, but these beneficial effects were abolished in *smn-1­(ok355);epg-5­(tm3425)* double mutants. (D-F) Resveratrol similarly improved neuromuscular function in *smn-1­(ok355)* animals but failed to rescue pumping or locomotory defects in the double mutant background. These findings indicate that the beneficial effects of rapamycin and resveratrol require an intact autophagy pathway. Data are presented as scatter plots and bar graphs with mean ± SEM; *n* > 25 animals per genotype per concentration across ≥ 3 independent trials; Two-Way ANOVA with Sidak’s multiple comparison test; ns = not significant, *p < 0.05, **p < 0.01, ***p < 0.001, and ****p < 0.0001.

To facilitate comparison across the compounds and experimental readouts, the principal findings are summarized in Table S2. Among the autophagy activators, rapamycin, resveratrol and torin-1 produced significant benefits across multiple SMA-associated phenotypes. Rapamycin at 50 μM, resveratrol at 100 μM and torin-1 at 500 nM represented the most favorable concentrations within their respective treatment groups, based on the consistency of improvement across assays and the effects observed in control animals. Overall, resveratrol at 100 μM displayed the most favorable profile, as it was the lowest concentration that consistently improved pharyngeal pumping, locomotion and survival, increased SMN-1 fluorescence and exhibited genetically demonstrated autophagy dependency, without adversely affecting neuromuscular function in control animals.

Overall, we provide the first evidence that pharmacological activation of autophagy enhanced neuromuscular performance in the severe *C. elegans* SMA model. While previous SMA mammalian studies have reported conflicting outcomes - some favoring autophagy inhibition and others activation^20,23–25,31,53^ - our data suggest that enhancing autophagic activity ameliorates major SMA-associated defects. More specifically, our results indicated that pharmacological activation of autophagy significantly ameliorated key disease-associated phenotypes in *smn-1­(ok355)* animals, including enhanced pharyngeal pumping (Figure 2), improved locomotor activity (Figure 3) and extended survival (Figure 4; Table S1). These beneficial effects were consistent across two severe *smn-1* mutant alleles and occurred independently of metabolically active food (Figure S2 and S3), indicating that our observed phenotypic improvements are not restricted to a specific genetic or dietary background. In contrast, autophagy inhibitors did not confer any benefit; notably, hydroxychloroquine treatment further reduced pharyngeal pumping rates in *smn-1­(ok355)* animals (Figure 1). Together, these findings indicate that the phenotypic rescue may be in part associated with increased SMN-1 protein abundance (Figure 5), while the loss of benefit in *smn-1­(ok355);epg-5­(tm3425)* double mutants demonstrates that an intact autophagic pathway is required for the neuromuscular improvement (Figure 6).

The beneficial effects observed were specific to *smn-1* deficiency, as no phenotypic improvements were evident in control animals (Figures 2A-C, 3–5, S2–S4), indicating a targeted therapeutic response. Although autophagy activators have been previously reported to extend *C. elegans* lifespan under certain conditions,^48,54^ we did not observe such effects in our control groups, further supporting the disease-specific nature of the therapeutic response in our SMA model. Furthermore, given that excessive autophagic activity has been associated with pharyngeal muscle damage in *C. elegans*,^55^ this mechanism could underlie the reduced pharyngeal pumping observed in control animals following treatment. These findings likely reflect a context-dependent balance between the protective and detrimental effects of autophagy activation.

Interestingly, V-ATPases, which are essential for lysosomal acidification and autophagic flux,^56^ are reportedly downregulated in *smn-1­(ok355)* animals.^57^ When autophagy initiation is intact, but flux is impaired, V-ATPase dysfunction may underlie this defect, with pharmacological autophagy activators potentially restoring proper autophagic activity.^30^ The beneficial outcomes observed herein may therefore arise from downstream effects on V-ATPases, possibly through the autophagy-related transcription factor EB (TFEB), which governs lysosomal and autophagic gene expression.^58^ Furthermore, prior studies reporting increased SMN protein levels and enhanced mitochondrial integrity following autophagy activation in mammalian models are consistent with our findings.^23,31,53^ In parallel, restoration of autophagic flux to basal levels has been shown to confer neuroprotection in SMA mice, further supporting a conserved therapeutic role for autophagy modulation across species.^59^ Consistent with these observations, we found that rapamycin and resveratrol significantly increased SMN-1 fluorescence intensity in *smn-1­(ok355)* animals (Figure 5). In contrast, the autophagy inhibitor 3-methyladenine did not affect SMN-1 abundance, whereas chloroquine further reduced SMN-1 fluorescence in the *C. elegans* SMA model (Figure 5). These findings suggest that the functional benefits of autophagy activation may be mediated, at least in part, through increased abundance or stability of the remaining SMN-1 protein. Nevertheless, the effects in control animals were compound dependent, with rapamycin reducing and resveratrol increasing SMN-1 fluorescence, respectively (Figure 5). Overall, our findings indicate that increased SMN-1 protein abundance is one mechanism underlying the therapeutic effects of these compounds; however, additional SMN-dependent and SMN-independent mechanisms may also be involved. Critically, the partial rescue of neuromuscular function following rapamycin and resveratrol treatment was abolished in *smn-1­(ok355);epg-5­(tm3425)* double mutant animals, indicating that the *smn-1* partial phenotypic rescue requires an intact autophagy pathway. As EPG-5 is required for late-stage autophagy and proper autophagic flux,^51,52^ these findings provide genetic evidence that the beneficial effects observed following treatment with rapamycin and resveratrol depend on an intact autophagic pathway rather than reflecting solely nonspecific pharmacological effects. When considered together with our SMN-1 fluorescence data, these results suggest that autophagy activation exerts therapeutic benefit in the SMA model through an autophagy-dependent mechanism that may, at least in part, promote or stabilize residual SMN-1 levels. More broadly, these findings strengthen the conclusion that enhancing autophagic activity is beneficial in this disease context.

From a translational perspective, autophagy activators could potentially be combined with SMN-restoring therapies, including antisense oligonucleotide-mediated *SMN2* splicing correction, small-molecule splicing modifiers or *SMN1* gene replacement. Such combinations would target complementary aspects of SMA pathology: SMN-directed therapies address the primary molecular deficiency, whereas autophagy activation may improve downstream proteostatic and lysosomal dysfunction and, based on our findings, increase or stabilize residual SMN protein. Among the compounds tested, we would prioritise 100 μM resveratrol for future preclinical cotreatment studies because it was the lowest concentration that consistently improved pharyngeal pumping, locomotion and survival, increased SMN fluorescence and exhibited *epg-5*-dependent functional activity without adversely affecting control neuromuscular function. Moreover, the effects of autophagy activation may differ according to disease severity and treatment stage. In a milder SMA model, the higher baseline level of neuromuscular function may produce smaller absolute improvements because of ceiling effects, with benefits instead appearing as delayed disease progression, preservation of function with age or increased survival. Conversely, a milder model may not display autophagic dysfunction, as has been noted in mice,^24^ and therefore enhancing autophagy may promote disease progression. As treatment in our study began during embryonic development, the current findings primarily model early intervention and do not establish whether autophagy activation would be beneficial under conditions of established degeneration.

Collectively, our study establishes pharmacological activation of autophagy as an effective strategy to ameliorate key disease-associated phenotypes in a well-characterized *C. elegans* model of SMA. The consistent improvements observed in pharyngeal pumping, locomotor activity, and survival in *smn-1* mutant animals highlight the functional relevance of autophagy modulation in this disease context. Importantly, given the high evolutionary conservation of autophagy and SMN-dependent pathways across species, these findings provide a biologically grounded framework that may inform translational efforts in mammalian systems. Together, these data demonstrate the therapeutic potential of targeting autophagic pathways as combinatorial treatment strategies for SMA.

## Methods

### *C. elegans* Strains and Maintenance

LM99 *smn-1­(ok355)­I/hT2­(I;III)*, HA2823 *smn-1­(rt248)­I/hT2­(I;III)*, DIM51 *smn-1­(ok355)/hT2I;+/hT2­[smn-1­(syb8399)­bli-4­(e937)­qIs48]­III*, DIM49 *+/hT2­(I;III);epg-5­(tm3425)­V* and DIM50 *smn-1­(ok355)­I/hT2­(I;III);epg-5­(tm3425)V C. elegans* strains were maintained at 20 °C on Nematode Growth Media (NGM) seeded with *OP50 Escherichia coli* as previously described.^60^ Heterozygous *smn-1­(ok355)/hT2* offspring, which are phenotypically indistinguishable from wildtype, were used as controls.^34^ Ethical approval was not required for this study because *C. elegans* is an invertebrate species not covered as a protected animal under the UK Animals (Scientific Procedures) Act 1986.

### Drug Plate Preparation

Drugs (Sigma-Aldrich) were mixed directly into molten NGM solution prior to plate pouring and subsequently plates were seeded with OP50 *Escherichia coli*. Concentrations listed in figures represent the final concentration of each drug within the NGM plate. Specifically, 3-methyladenine (#M9281) was dissolved in sterile distilled water at concentrations of 0 μM, 33 μM, 165 μM and 330 μM. Chloroquine (#C6628) was dissolved in dimethyl sulfoxide (DMSO) at concentrations of 0 μM, 33 μM, 165 μM and 250 μM. Hydroxychloroquine (#H0915) was dissolved in DMSO at concentrations of 0 μM, 33 μM, 100 μM and 165 μM. Wortmannin (#W1628) was dissolved in DMSO at concentrations of 0 nM, 10 nM, 50 nM and 100 nM. Rapamycin (#553210) was dissolved in DMSO at concentrations of 0 μM, 1 μM, 50 μM and 100 μM. Resveratrol (#554325) was dissolved in DMSO at concentrations of 0 μM, 50 μM, 100 μM and 200 μM. Torin-1 (#475991) was dissolved in DMSO at concentrations of 0 nM, 250 nM, 500 nM and 1000 nM. Spermidine (#05292) was dissolved in sterile distilled water at concentrations of 0 μM, 33 μM, 165 μM and 330 μM.

### Generation and Treatment of Synchronized Animals

Age-synchronized *C. elegans* progeny was generated by a timed egg-laying assay. Adult parental animals were transferred to freshly prepared NGM plates containing either vehicle or the drug and allowed to lay eggs for 5 h. The parents were then removed, and the resulting progeny remained on the same vehicle- or drug-containing plates throughout development. Overall, both the experimental and their parental animals were exposed to the assigned treatment condition, with progeny exposure commencing at the embryonic stage.

For all experiments, “day 3” refers to animals analyzed at the midpoint of the 5-h egg lay after hatching at 25 °C for 2 days and 20 °C for 1 day. This paradigm has been previously selected because *smn-1­(ok355)* animals exhibit their established late-larval disease phenotype at day 3 posthatching and arrest around the L4/final larval stage, as previously described.^40^

### Neuromuscular Assays

The pharyngeal pumping assay was performed as previously described,^36^ and locomotion was assessed using a Zeiss Discovery.V8 SteREO microscope with a 63× objective and AxioCam ICc5 camera (15 frames/sec). Animals were recorded for 5 min and analyzed using WormLab 1.1 software (MBF Bioscience). For experiments involving metabolically inactive food, OP50 *E. coli* was heated to 65 °C for 30 min^61^ and concentrated 5x prior to seeding NGM plates. Each experiment consisted of *n* ≥ 3 independent trials (*n* > 25 animals per genotype, per concentration), with at least one trial blinded.

### Lifespan Assays

Lifespan assays were performed at 20 °C as previously described.^34^ Briefly, animals were reared on plates containing vehicle or drug and transferred to NGM plates on day 3 without the use of 5′-fluorodeoxyuridine (FUdR) due to its effect on *C. elegans* lifespan.^62^ Instead, animals were transferred to fresh NGM plates every 2 days. Nematodes were scored daily and censored for loss, intestinal rupture, internal hatching or crawling off the plate. Each condition included ≥ 90 animals and was repeated in *n* ≥ 3 independent trials, with at least one trial blinded.

### SMN-1 Fluorescence Quantification

DIM51 animals carrying an endogenous C-terminal wrmScarlet tag on the wildtype *smn-1* copy located on the *hT2* balancer chromosome were originally generated by Sunybiotech using CRISPR/Cas9 editing and backcrossed two times. Accordingly, wrmScarlet-tagged SMN-1 fluorescence was quantified as a proxy for SMN-1 protein abundance in both control and *smn-1­(ok355)* animals.

Stage matched-control and *smn-1­(ok355)* animals were exposed to vehicle or the indicated concentrations of 3-methyladenine, chloroquine, rapamycin or resveratrol from the embryonic stage, as described above. Day 3 animals were mounted on 2% agar pads and immobilized using 30 mg/mL 2,3-Butanedione monoxime. SMN-1 fluorescence was captured using a Zeiss AxioImager.M2 equipped with an Axiocam 503 camera with a 10x objective. Images within each independent experiment were acquired using identical exposure, gain and illumination settings. Mean fluorescence intensity was quantified within the entire animal using Zeiss Zen 3.3 software. Fluorescence intensity is reported in arbitrary units. Each condition included *n* > 25 animals across 3 independent trials, with at least one experiment performed under blinded conditions.

### Statistical Analysis

Statistical analyses were performed using GraphPad PRISM [version 10.3.1]. Outliers were identified and removed using Grubb’s test. Data were analyzed using two-way ANOVA with Sidak’s multiple comparison’s test, whereas Kaplan–Meier survival curves were assessed by log-rank test. Statistical significance was set at *p < 0.05*, with significance levels indicated as ns = not significant, *p < 0.05, **p < 0.01, ***p < 0.001, and ****p < 0.0001.

## Supplementary Material



## Notes

Representative bright-field images of age-matched control and *smn-1­(ok355)* animals; pharyngeal pumping responses to autophagy activators in the alternative severe *smn-1­(rt248)* mutant allele; pharyngeal pumping responses to autophagy activators under metabolically inactive food conditions; lifespan analyses of control animals treated with autophagy activators; time-course survival percentages for *smn-1­(ok355)* animals following rapamycin, resveratrol, and torin-1 treatment; summary of the effects of pharmacological autophagy modulators across phenotypic and mechanistic readouts (PDF)

## Abbreviations

SMA: spinal muscular atrophy
SMN: survival motor neuron
*SMN1*: *survival motor neuron 1*
*SMN2*: *survival motor neuron 2*
V-ATPase: Vacuolar ATPase
