# Supplementary material for: Pharmacological Activation of Autophagy Ameliorates Neuromuscular-Associated Defects and Extends Lifespan in a Caenorhabditis elegans Model of Spinal Muscular Atrophy
Source: ACS Chem Neurosci. 2026 Sep 2;17(18):3272–82. doi: 10.1021/acschemneuro.6c00569 (PMC13587599; doi:10.1021/acschemneuro.6c00569)
Supplement: Supplementary file 1 [file cn6c00569_si_001.pdf]

*Supporting Information for*

**Pharmacological Activation of Autophagy Ameliorates Neuromuscular-Associated Defects and Extends Lifespan in a *Caenorhabditis elegans* Model of Spinal Muscular Atrophy**

Saman Rashid[1], Coral Crespo Gómez[1] and Maria Dimitriadi[1*]

[1]University of Hertfordshire, School of Health, Medicine and Life Sciences, Hatfield, England, UK AL10 9AB

*Corresponding author: Dr Maria Dimitriadi, **m.dimitriadi@herts.ac.uk**

# Figure S1

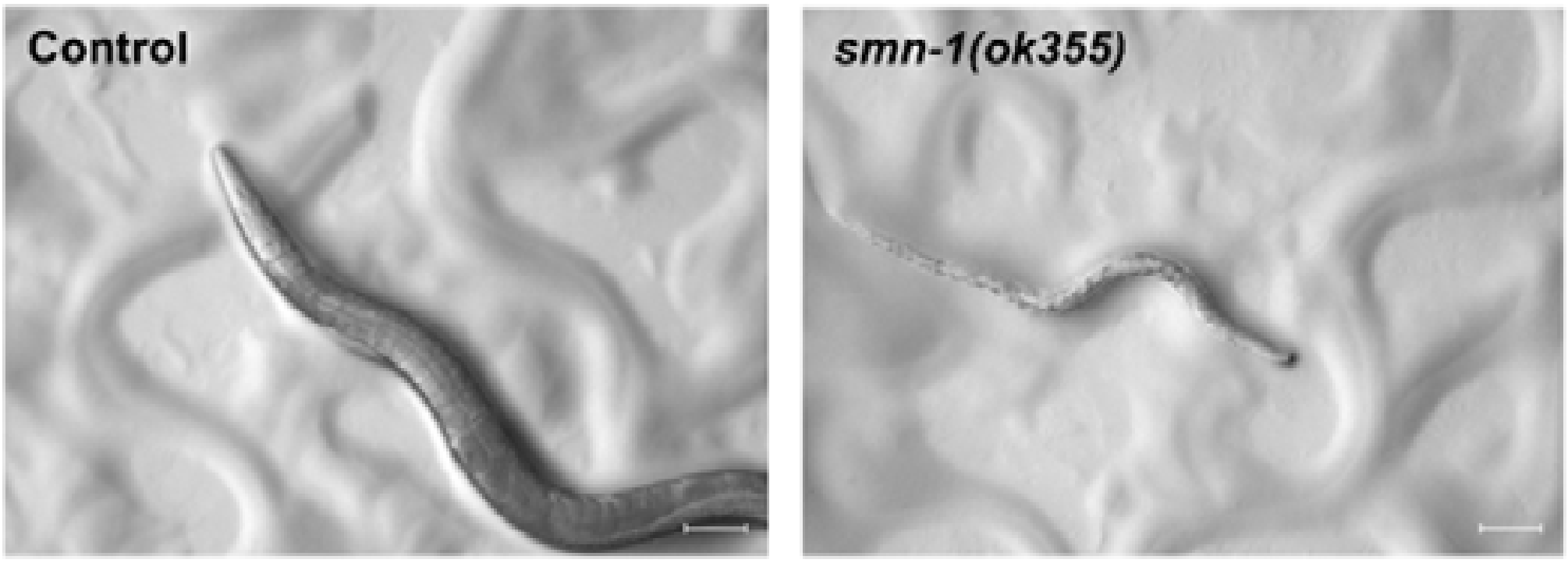


**Figure S1. SMN-1 deficiency results in marked phenotypes in *C. elegans*.** Representative bright-field images of age-matched control and homozygous *smn-1(ok355)* animals at day 3 post-hatching. At this time point, *smn-1(ok355)* animals display reduced body size, developmental arrest and neuromuscular defects. Scale bars = 200 µm.

# Figure S2

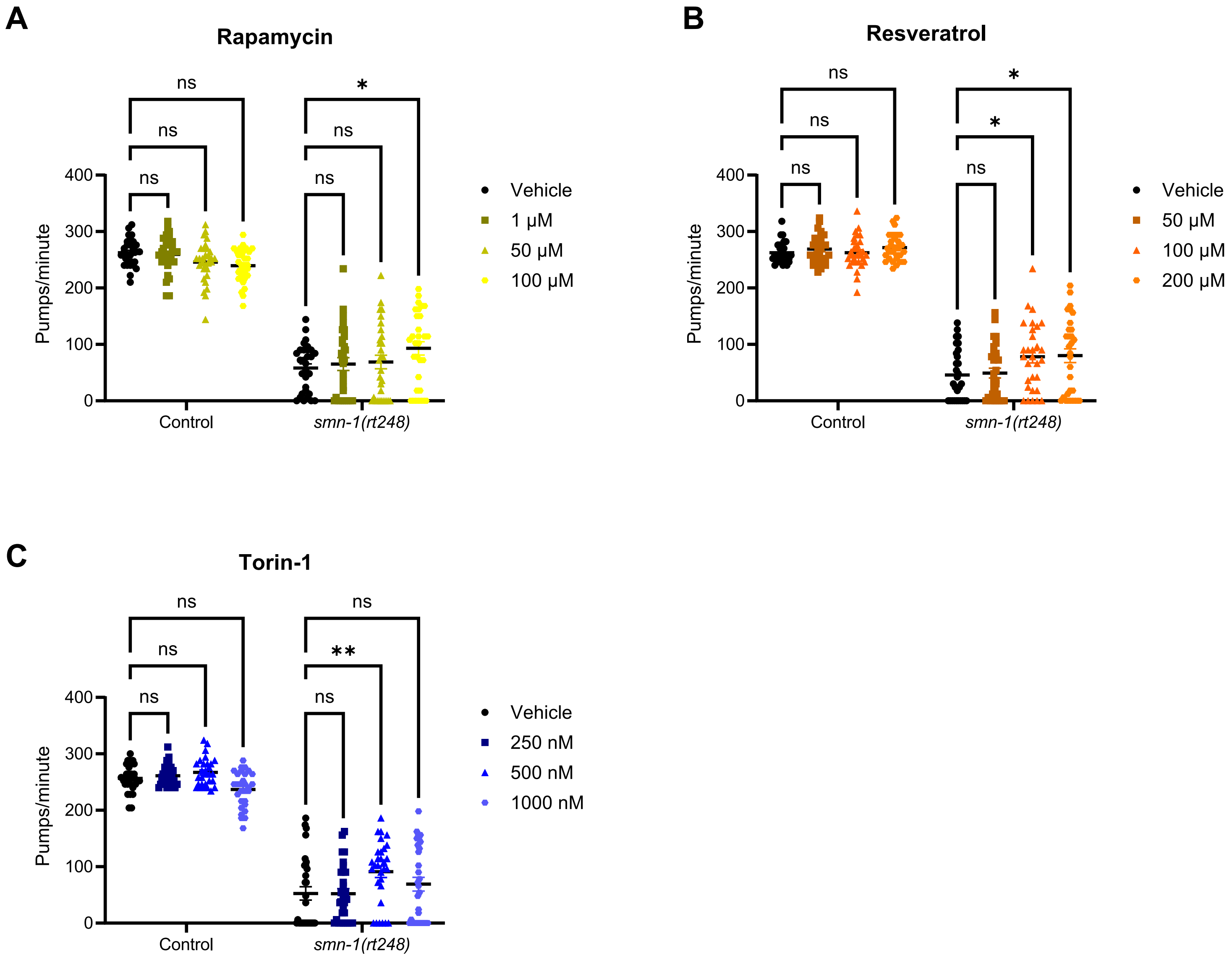


**Figure S2. Autophagy activators improve neuromuscular function by pharyngeal pumping in an alternative severe *smn-1(rt248)* mutant allele.** *smn-1(rt248)* and control animals were reared on NGM plates containing either vehicle or varying concentrations of autophagy activators. Animals were assessed at Day 3 post hatching. (A) Rapamycin improved pharyngeal pumping in *smn-1(rt248)* animals at 100 μM ($p$ = *0.0239*). (B) Resveratrol improved pharyngeal pumping in *smn-1(rt248)* animals at 100 μM ($p$ = *0.0207*) and 200 μM ($p$ = *0.0128*). (C) Torin-1 improved pharyngeal pumping in *smn-1(rt248)* animals at 500 nM ($p$ = *0.0074*). Data shown as a scatter plot, where error bars represent the ± SEM; n > 25 animals per genotype per concentration across ≥ 3 independent trials; Two-Way ANOVA with Sidak's multiple comparison test; ns = not significant, *$p < 0.05$ and **$p < 0.01$.

# Figure S3

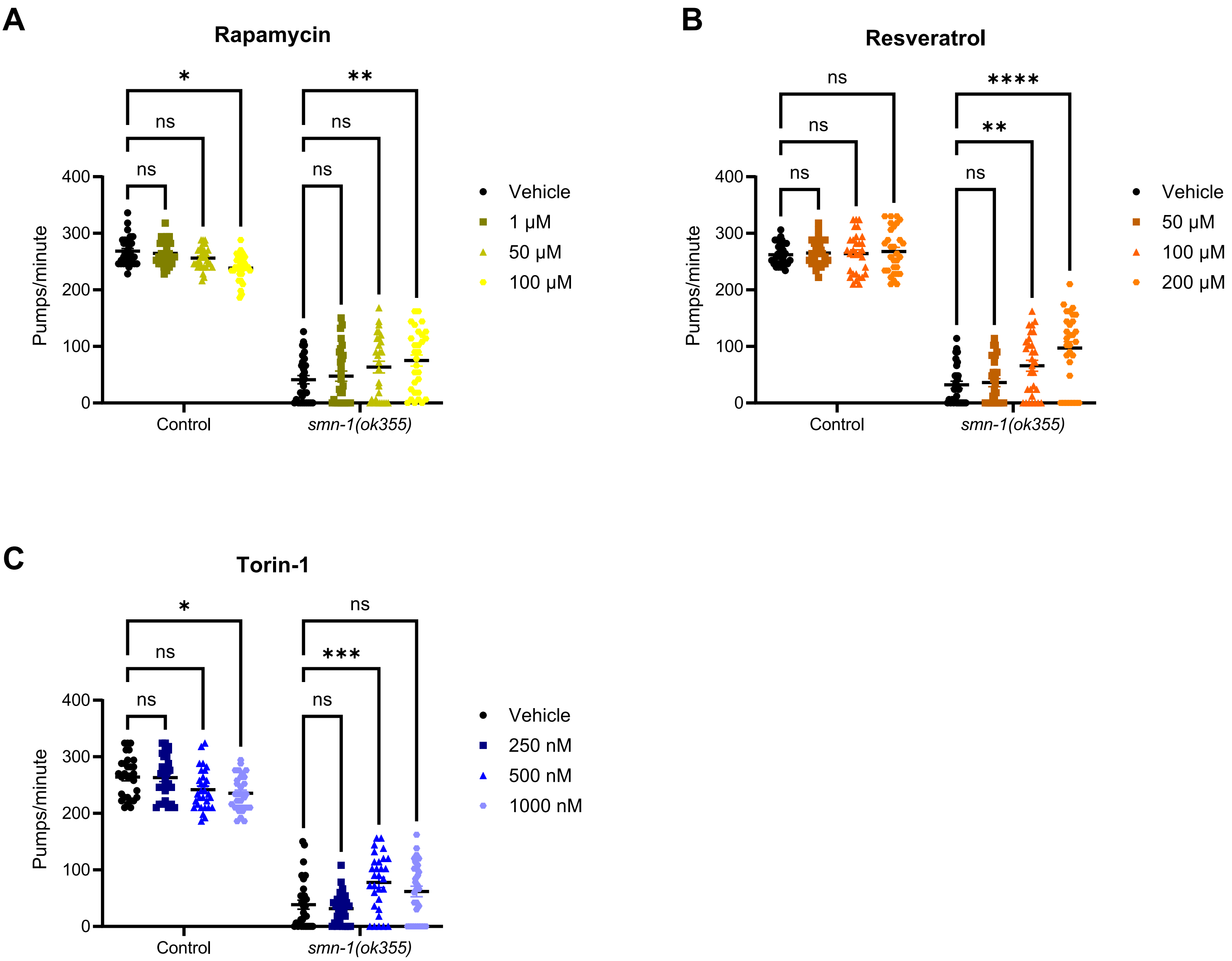


**Figure S3. Autophagy activators improve pharyngeal pumping under metabolically inactive food conditions in *smn-1(ok355)* animals.** *smn-1(ok355)* and control animals were reared on NGM plates containing either vehicle or varying concentrations of autophagy activators and seeded with metabolically inactive *OP50 E. coli*. Animals were assessed at Day 3 post hatching. Pharyngeal pumping was defined as a complete contraction and relaxation of the grinder. (A) Rapamycin reduced pharyngeal pumping in control animals at 100 μM ($p = 0.0160$) but improved pharyngeal pumping in *smn-1(ok355)* mutants reared on metabolically inactive food ($p = 0.0048$). (B) Resveratrol improved pharyngeal pumping of *smn-1(ok355)* animals at 100 μM ($p = 0.0092$) and 200 μM ($p < 0.0001$). (C) Torin-1 reduced pharyngeal pumping in control animals at 1000 nM ($p = 0.0279$) and partially rescued pharyngeal pumping in *smn-1(ok355)* animals reared on metabolically inactive food at 500 nM ($p = 0.0010$). Data shown as a scatter plot, where error bars represent the ± SEM; n > 25 animals per genotype per concentration across ≥ 3 independent trials; Two-Way ANOVA with Sidak's multiple comparison test; ns = not significant, $*p < 0.05$, $**p < 0.01$, $***p < 0.001$, and $****p < 0.0001$.

# Figure S4

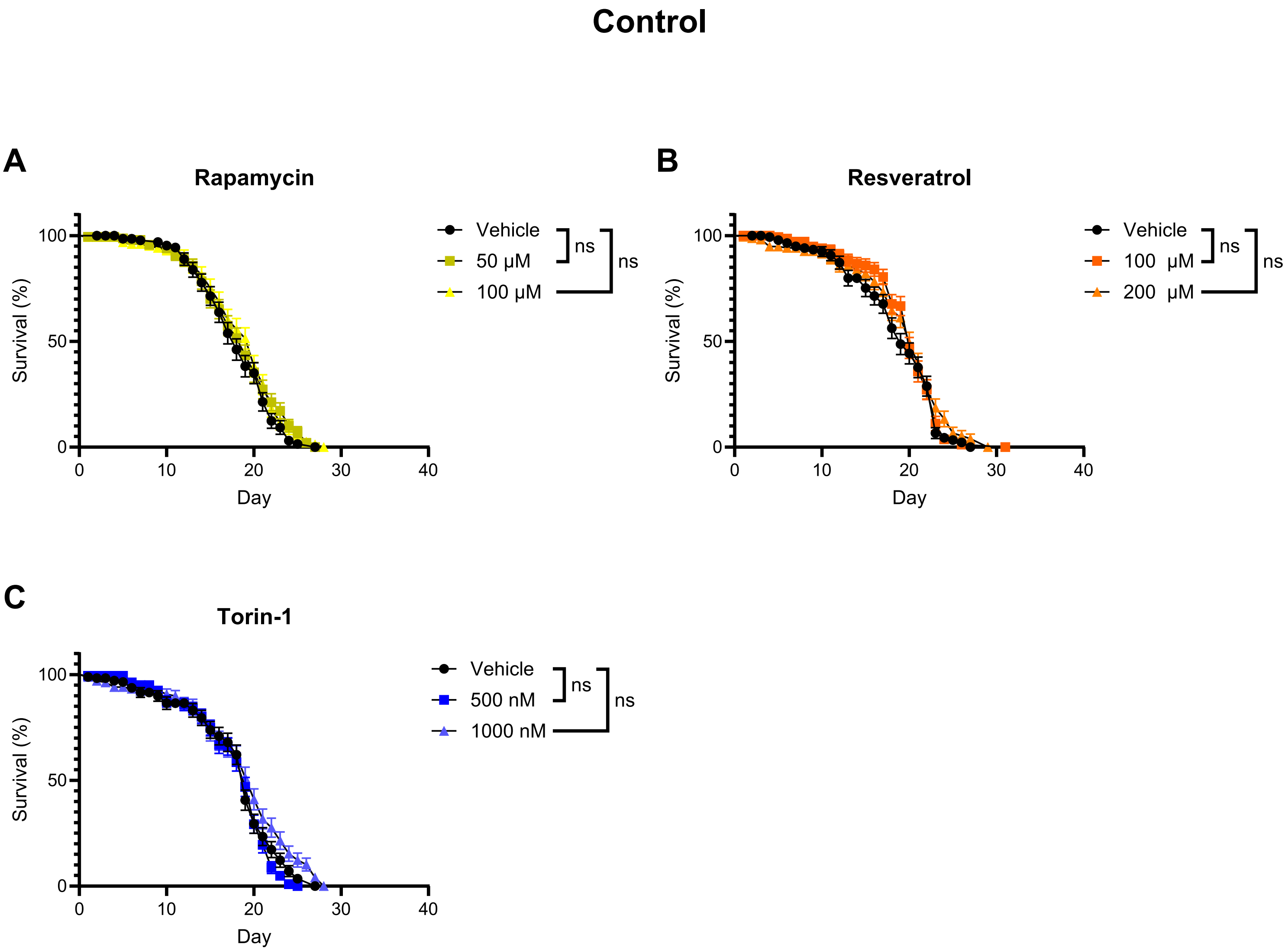


**Figure S4. Autophagy activators have no effect on control animal lifespan.** Control animals were reared on NGM plates containing either vehicle or varying concentrations of autophagy activators. Animals were age-matched and transferred to regular NGM plates at Day 3. Animals were then scored for survival daily and censored for loss, internal hatching, or climbing out of the plate. (A) Rapamycin had no significant effect on control animal lifespan. (B) Resveratrol had no significant effect on control animal lifespan. (C) Torin-1 had no significant effect on control animal lifespan. Data shown as Kaplan-Meier survival curves, where error bars represent the standard error of the survival estimate; ≥ 90 per concentration across ≥ 3 independent trials; log-rank test; ns = not significant.

**Table S1. Time-course survival table showing the percentage of surviving *smn-1(ok355)* animals at each day for each treatment group.**

| Day | Rapamycin Vehicle (%) | Rapamycin 50 µM (%) | Rapamycin 100 µM (%) | Resveratrol Vehicle (%) | Resveratrol 100 µM (%) | Resveratrol 200 µM (%) | Torin-1 Vehicle (%) | Torin-1 500 nM (%) | Torin-1 1000 nM (%) |
|---|---|---|---|---|---|---|---|---|---|
| 0 | 100.00 | 100.00 | 100.00 | 100.00 | 100.00 | 100.00 | 100.00 | 100.00 | 100.00 |
| 1 | 97.54 | 99.08 | 100.00 | 99.25 | 99.25 | 100.00 | 100.00 | 97.30 | 99.32 |
| 2 | 95.06 | 97.23 | 96.12 | 93.98 | 96.18 | 95.62 | 93.24 | 88.51 | 96.58 |
| 3 | 86.79 | 92.60 | 89.25 | 89.47 | 91.56 | 93.43 | 90.54 | 85.11 | 86.85 |
| 4 | 75.22 | 87.92 | 81.40 | 81.13 | 87.68 | 89.05 | 83.52 | 79.62 | 76.43 |
| 5 | 52.40 | 72.80 | 62.54 | 44.82 | 67.33 | 72.86 | 53.34 | 68.54 | 63.23 |
| 6 | 25.36 | 41.60 | 31.77 | 19.92 | 43.84 | 49.85 | 18.25 | 45.00 | 34.42 |
| 7 | 4.37 | 15.48 | 13.90 | 4.98 | 18.79 | 24.54 | 0.73 | 11.08 | 9.13 |
| 8 | 0.00 | 4.84 | 2.98 | 0.00 | 5.48 | 6.54 | 0.00 | 4.43 | 3.32 |
| 9 | 0.00 | 0.97 | 0.00 | 0.00 | 0.00 | 0.00 | 0.00 | 0.74 | 0.00 |
| 10 | 0.00 | 0.00 | 0.00 | 0.00 | 0.00 | 0.00 | 0.00 | 0.00 | 0.00 |

**Table S2. Summary of the effects of pharmacological autophagy modulators in the *C. elegans smn-1(ok355)* SMA model**

| Compound | Doses assessed | Pharyngeal pumping | Locomotion | Lifespan | SMN-1 fluorescence | Autophagy dependency | Overall interpretation |
|---|---|---|---|---|---|---|---|
| **3-Methyladenine** | 33, 165 and 330 μM | No improvement | ND | ND | No significant change at 165 or 330 μM | ND | No beneficial dose identified |
| **Chloroquine** | 33, 165 and 250 μM | No improvement | ND | ND | Reduced at 165 and 250 μM | ND | No benefit; further reduced SMN-1 abundance |
| **Hydroxychloroquine** | 33, 100 and 165 μM | No improvement; worsened pumping at 100 μM | ND | ND | ND | ND | Not suitable for further investigation |
| **Wortmannin** | 10, 50 and 100 nM | No improvement | ND | ND | ND | ND | No beneficial dose identified |
| **Rapamycin** | 1, 50 and 100 μM | Improved at 1, 50 and 100 μM | Improved at 50 and 100 μM | Increased survival at 50 and 100 μM | Increased at 50 and 100 μM | Rescue lost in *smn-1;epg-5* animals | 50 μM most favourable within this compound, but adverse effects were observed in controls |
| **Resveratrol** | 50, 100 and 200 μM | Improved at 100 and 200 μM | Improved at 100 and 200 μM | Increased survival and median lifespan at 100 and 200 μM | Increased at 100 and 200 μM | Rescue lost in *smn-1;epg-5* animals | 100 μM preferred overall: lowest consistently effective dose with no adverse functional effect in controls |
| **Torin-1** | 250, 500 and 1000 nM | Improved at 500 nM | Improved at 500 and 1000 nM | Increased survival at 500 and 1000 nM | ND | ND | 500 nM most favourable within this compound; higher dose affected controls |
| **Spermidine** | 33, 165 and 330 μM | Improved in *smn-1* animals, but also improved controls | ND | ND | ND | ND | Not pursued because the effect was not SMA-specific |

**Abbreviations:** ND, not determined.
